# Isoniazid- and ethambutol-induced psychosis

**DOI:** 10.4103/1817-1737.43083

**Published:** 2008

**Authors:** Prasad R., Rajiv Garg, Sanjay Kumar Verma

**Affiliations:** *Department of Pulmonary Medicine, CSM Medical University, UP, Lucknow - 226 003, India*

**Keywords:** Ethambutol, isoniazid, psychosis

## Abstract

Most cases of antituberculous agent–associated psychoses were caused by isoniazid (INH), with ethambutol (EMB)-induced psychosis being rare. The concomitant occurrence of INH- and EMB- induced psychosis and in a single individual is extremely uncommon. We report a case of 28-year-old male who developed psychotic symptoms on start of EMB initially and later on INH also. He was prescribed rifampicin, pyrazinamide, and ofloxacin and had no further psychotic symptoms.

Isoniazid (INH) is included in all drug regimens used for the treatment of tuberculosis because of its potency, safety and low cost. The most common adverse reactions observed with INH are peripheral neuropathy and hepatitis. In our review of the literature, we found that most cases of antituberculous agent–associated psychoses were caused by INH, with ethambutol (EMB)-induced psychosis being rare. The concomitant occurrence of INH- and EMB-induced psychosis and in a single individual, an extremely uncommon event, prompted us to report this case.

## Case Report

A 28-year-old male, a nonsmoker, was admitted to our department with the complaints of recurrent hemoptysis and loss of appetite for 1 month. His past history was not significant. His resting pulse rate was 102/min and blood pressure was 112/74 mm Hg and his respiratory rate was 26/min. His general examination revealed no significant abnormality. His respiratory system examination revealed bilateral coarse crepitations. He was given symptomatic treatment for the hemoptysis and a chest x-ray was taken, which showed infiltrative lesions confined to the upper zones of both lungs.

His blood examination revealed a total leucocyte count of 10,200/cmm; the differential count showed 66% polymorphs and 34% lymphocytes. His PPD showed 30 mm induration. Sputum tested on three consecutive days was positive for AFB. He was prescribed short-course antituberculous treatment as per WHO guidelines, with an initial 2 months of rifampicin, isoniazid, ethambutol, and pyrazinamide followed by 4 months of rifampicin and isoniazid. The first 3 days of treatment with this four-drug regimen was uneventful. On the fourth day, however, the patient suddenly became restless, irritable, and agitated, with aimless, incongruous acts, and irrelevant talking; he also started having visual hallucinations. He had no past history of any mental illness. There was no neurological deficit and fundus examination was normal (as per the neurology evaluation). An initial diagnosis of drug-induced psychosis was made after a psychiatric consultation, with isoniazid being identified as the likely culprit. The patient's symptoms responded to an injection of diazepam. All the antituberculous drugs were withdrawn. The patient became free of psychotic symptoms over the next 2 days. Antituberculous therapy was now restarted, with ethambutol, rifampicin, and pyrazinamide being added one by one, starting with a low ‘test’ dose of each [Table 1]. We avoided isoniazid initially since we were fairly certain that it was the offending agent. As can be seen from the table, psychotic symptoms were seen 5 days after ethambutol was started, disappeared after the drug was stopped, and recurred when it was restarted. Similarly, we were able to show that isoniazid could also independently bring on the symptoms and that withdrawal of the drug led to remission of symptoms.

**Table 1:** The reintroduction schedule of antituberculous drugs in present case report

Days	Drug given	Appearance of psychotic symptoms
Day 1	Ethambutol 300 mg	No
Day 2	Ethambutol 600 mg	No
Day 3	Ethambutol 800 mg	No
Day 4	Rifampicin 150 mg + ethambutol 800 mg	No
Day 5	Rifampicin 300 mg + ethambutol 800 mg	Appearance of psychotic symptoms
Day 6	All drugs stopped	Psychotic symptoms persisted
Day 7	All drugs stopped	Psychotic symptoms resolved
Day 8	Ethambutol 300 mg	No
Day 9	Ethambutol 600 mg	
	Thus, the ethambutol could be proved to be the responsible for the causation of the psychotic symptoms and could not be reintroduced.	Appearance of psychotic symptoms
Day 10	All drugs stopped till the resolutions of psychotic symptoms.	Psychotic symptoms persisted
Day 11	No ATT given	Psychotic symptoms resolved
Day 12	Rifampicin 150 mg	No
Day 13	Rifampicin 300 mg	No
Day 14	Rifampicin 450 mg	No
Day 15	Rifampicin 450 mg+ pyrazinamide 250mg	No
Day 16	Rifampicin 450 mg+ pyrazinamide 500mg	No
Day 17	Rifampicin 450 mg+ pyrazinamide 1500mg	No
Day 18	Day 16 regimen + isoniazid 50 mg	No
Day 19	Day 16 regimen + isoniazid 300 mg	
	Thus, isoniazid was also proved to be responsible for the causation of the psychotic symptoms and could not be reintroduced.	Appearance of psychotic symptoms again
Day 20	Day 16 regimen	Psychotic symptoms persisted
Day 21	Day 16 regimen continued	Psychotic symptoms resolved

Thus, both isoniazid and ethambutol proved to be responsible for his psychotic behavior and had to be stopped. He was, instead, prescribed rifampicin, pyrazinamide, and ofloxacin and had no further psychotic symptoms.

## Discussion

Psychiatric disorders have traditionally been considered to be mental rather than physical illnesses. This is because they manifest with disordered functioning in the areas of emotions, perceptions, thinking, and memory, and/or have no established biological basis. Psychiatric disorders are diagnosed primarily by recognizing the pattern of symptoms. The term psychosis is used to described illness in which the patient has altered perception of reality as evidenced by delusions and or hallucination

Our review of literature showed that most cases of antituberculous drug–associated psychoses were due to isoniazid; ethambutol-induced psychosis is rare and the concomitant occurrence in an individual of both isoniazid- and ethambutol-induced psychosis, as seen in the present case, is extremely rare.

Isoniazid-related psychiatric disorders reported in the literature include psychosis, obsessive-compulsive neurosis, and mania.[1] Loss of memory and death following ingestion of isoniazid has also been reported.[2–3]

The first description of psychotic symptoms due to isoniazid was by Mandel *et al.*, who reported three such cases in 1956.[4]

The mechanism of production of isoniazid-related psychiatric disorders is not clearly known, but isoniazid is known to interfere with several metabolic processes essential for the normal functioning of the neuron.[5] Isoniazid causes deficiency of vitamin B_6_ by causing excessive excretion of the vitamin, which in turn leads to a disturbance of normal tryptophan metabolism. Isoniazid also inhibits the activity of brain pyridoxal-5-phosphate (produced in the body from pyridoxine), which leads to a decrease in brain gamma-aminobutyric acid and other synaptic transmitters, resulting in neurologic ill effect.[6] Other predisposing factors for the occurrence of psychotic illness are diabetes mellitus, hepatic insufficiency, old age, alcoholism, and family and personal history of mental illness. Incidentally, no such risk factors were seen in our patient.

There is great variability in the clinical features of isoniazid-induced psychosis in the various reported cases. Jackson, in 1957, reported five cases of isoniazid-induced psychosis that presented with excessive argumentation, mental depression, euphoria, grandiose ideas, and complex delusions; none of these patients had any previous history of mental illness.[7] Agarwala, in 1974, reported symptoms of restlessness, irritability, emotional instability, agitation, apprehension, and fluctuation in behavior after isoniazid therapy[8] (as was seen in our patient). Bedi, in 1994, reported a case of isoniazid psychosis in a 74-year-old, who developed restlessness, irritability, aimless activity, and incongruous actions 10 days after starting isoniazid therapy.[9] In 1996, Tiwari reported a case of isoniazid-induced psychosis with disturbed sleep, restlessness, and abnormal behavior[10] (as in the present case). The durations of psychotic symptoms in these case reports varied widely: i.e., 7–45 days,[7] 7 days,[8] 10 days,[9] and 120 days.[10]

Ethambutol is one of the most commonly used drugs in the treatment of tuberculosis. The principle side effect is retrobulbar neuritis. Central nervous system toxicity is not widely reported and psychosis secondary to it is very rare.[11] The exact mechanism of ethambutol-induced psychosis is not clear. The symptomatology of ethambutol-induced psychosis is almost same as that of isoniazid. Hsu in 1999 reported a case of ethambutol-related psychosis where there were symptoms like dizziness, disorientation, and auditory and visual hallucinations after 7 days of ethambutol intake.[12]

The diagnostic criteria for substance-induced psychosis, as per as DSM IV classification, are given below:

- Prominent hallucinations or delusions
- There is evidence from the history, physical examination, or laboratory findings of either (1) or (2):

1. The symptoms in criterion A develop during, or within a month of, substance intoxication or withdrawal
2. Medication use is etiologically related to the disturbance C. The symptoms precede the onset of the substance use (or medication use); the symptoms persist for a substantial period of time (e.g., about a month) after the cessation of acute withdrawal or severe intoxication, or are substantially in excess of what would be expected given the type or amount of the substance used or the duration of use; or there is other evidence that suggests the existence of an independent non-substance-induced psychotic disorder (e.g., a history of recurrent non-substance-related episodes).

With regard to management, it is known that acute psychosis induced by isoniazid and ethambutol tends to subside once the precipitating stresses are over or their intensity is reduced. Thus, patients with isoniazid induced-psychosis recover without specific treatment after the withdrawal of the offending drug. The administration of pyridoxine, which has been advocated for the prevention and treatment of isoniazid-induced neurologic manifestations,[1] failed to achieve desired results in isoniazid-induced psychosis.

In the present case, following discontinuation of all antituberculous agents, the psychiatric symptoms subsided. When the patient was challenged with isoniazid and ethambutol, the same psychiatric symptoms recurred, but resolved again after discontinuation of the drugs.

## Conclusion

It is important to be aware that both isoniazid and ethambutol can induce psychosis concomitantly in an individual when antitubercular medications are prescribed.
